# Thermal Reactivity in Metal Organic Materials (MOMs): From Single-Crystal-to-Single-Crystal Reactions and Beyond

**DOI:** 10.3390/ma12244088

**Published:** 2019-12-07

**Authors:** Javier Martí-Rujas

**Affiliations:** 1 Dipartimento di Chimica Materiali e Ingegneria Chimica. “Giulio Natta”, Politecnico di Milano, Via L. Mancinelli 7, 20131 Milano, Italy; javier.marti@polimi.it; Tel.: +39-02-2399-3047; 2 Center for Nano Science and Technology@Polimi, Istituto Italiano di Tecnologia, Via Pascoli 70/3, 20133 Milano, Italy

**Keywords:** high temperature solid-state chemistry, coordination polymers, metal-organic materials (MOMs), second-sphere coordination, single crystal X-ray diffraction, ab initio powder X-ray diffraction, single-crystal-to-single-crystal, crystal-to-polycrystalline transformation, crystal-to-amorphous-to-polycrystalline transformation

## Abstract

Thermal treatment is important in the solid-state chemistry of metal organic materials (MOMs) because it can create unexpected new structures with unique properties and applications that otherwise in the solution state are very difficult or impossible to achieve. Additionally, high-temperature solid-state reactivity provide insights to better understand chemical processes taking place in the solid-state. This review article describes relevant thermally induced solid-state reactions in metal organic materials, which include metal organic frameworks (MOFs)/coordination polymers (CPs), and second coordination sphere adducts (SSCs). High temperature solid-state reactivity can occur in a single-crystal-to-single crystal manner (SCSC) usually for cases where there is small atomic motion, allowing full structural characterization by single crystal X-ray diffraction (SC-XRD) analysis. However, for the cases in which the structural transformations are severe, often the crystallinity of the metal-organic material is damaged, and this happens in a crystal-to-polycrystalline manner. For such cases, in the absence of suitable single crystals, structural characterization has to be carried out using ab initio powder X-ray diffraction analysis or pair distribution function (PDF) analysis when the product is amorphous. In this article, relevant thermally induced SCSC reactions and crystal-to-polycrystalline reactions in MOMs that involve significant structural transformations as a result of the molecular/atomic motion are described. Thermal reactivity focusing on cleavage and formation of coordination and covalent bonds, crystalline-to-amorphous-to-crystalline transformations, host–guest behavior and dehydrochlorination reactions in MOFs and SSCs will be discussed.

## 1. Introduction

Unlike in the gas phase and in the solution state, reactivity in the solid state is much more limited because molecular/atomic motion is constrained by the 3D arrangement in the crystalline lattice [1]. Solid-state reactivity in chemistry is very attractive because the products obtained can be unique since using solution chemistry might result in completely different products. Using the restricted environment of the crystalline state, by using defined distances and orientations for an efficient orbital overlapping of the reactive moieties it is possible to control the products. In other words, solid-state reactions (i.e., ethylenic bonds→cyclobutanes) can be very selective if the reacting groups are suitably placed in the solid material. Another important aspect to highlight is that in solid-state chemistry no solvent is used in the preparation of the material thus contributing to the green chemistry development that aims to a sustainable renewable chemistry.

The origin of solid-state reactivity in organic materials was conceived by Schmidt and co-workers in the 1960s where through the topochemical postulate it was proposed that solid-state reactions could proceed with the minimum amount of molecular motion [2,3,4]. In this regard, one aspect to consider if one reaction can be classified as topochemical is that at least one of the lattice axis in the unit cell should remain unaltered. As a result of the topochemical control, regio- and stereo-selective products can be obtained with considerable control and high conversion rates [5]. This was the starting point to establish the fields of organic solid-state and crystal engineering by careful selection of molecules with reactive functional groups that could crystallize and further react upon external stimuli [6,7,8].

The use of external stimuli to induce solid state reactivity can be studied by different approaches such as light induced reactivity [9,10,11,12,13], mechanical reactivity [14,15,16], high pressure reactivity [17,18], thermally induced reactions [19,20,21,22], or a combination of temperature and other aspects that might influence the reaction [23]. High temperature synthesis in MOMs is very appealing as often many synthesized materials obtained as kinetic products can be transformed into more stable thermodynamic structures upon heating. Crucially, the observation of the structural transformations induced by heat (i.e., kinetic to thermodynamic products) is very important to understand the reactivity of a given material. In such cases, if the thermally induced products can be analyzed by single crystal X-ray diffraction, structure-properties as a function of temperature can be established. It is important to note that there are many cases in which the products resulting from heating, undergo severe structural changes, and those products are usually not obtained as single crystals but as microcrystalline products (i.e., increase of mosaicity, crystal decay, and amorphization processes). Therefore, direct structure determination from single crystal X-ray diffraction where the products are microcrystalline is very difficult if not impossible.

The lack of a single crystal is a major problem that researchers must face if the target is to fully understand the atomic arrangement after the solid-state reaction, and alternative approaches enabling the full 3D structural description need to be sought. In such cases, ab initio powder XRD needs to be performed, although the process of solving a structure form powder X-ray diffraction data is much more complicated than single crystal X-ray diffraction [24]. However, there has been an important progress in the techniques that allow full structure elucidation from powdered materials [25,26,27]. Particularly, using the latest advances in ab initio powder X-ray structural analysis numerous challenging structures, including highly flexible organic molecules (peptides) [28,29], but also MOFs have been elucidated providing valuable structural information [30,31,32,33]. In fact, to our best knowledge, full structure determination from powder X-ray diffraction data on MOMs describing major structural transformations induced by temperature are still very limited.

Another scenario that often occurs when thermal reactions are being carried out in MOMs is that in some cases amorphous phases are formed. Because the structure of the amorphous phase is very important for the formation of new structures (i.e., acting as a precursors), alternative diffraction techniques are being used, such as pair distribution function (PDF) analysis [34]. As amorphous phases do not give sharp Bragg diffraction peaks, but diffuse scattering containing crucial information on two-atom interactions PDF analysis can be used to obtain short to medium range ordering. Thus, a map of distances between pairs of atoms can be obtained using the pair distribution function which gives information on the degree of local and intermediate ordering in amorphous materials.

The aim of this review is to put together relevant publications in which significant transformations in MOMs induced by temperature are reported. Such heat induced reactions can include severe transformations involving SCSC processes but also solid-state reactions that go beyond the single crystal-to-single-crystal limit and occur via a crystal-to-polycrystalline manner. In some cases, intermediate amorphous phases are also formed due to large structural transformations such as sliding of frameworks or cleavage and formation of chemical bonds. There are numerous reviews on SCSC published recently, where the interested reader can get an exhaustive description of structural transformations in the solid-state that cover not only heat as external stimuli [35,36,37,38].

## 2. Hybrid Metal Organic Materials: Coordination Polymers (CPs)/Metal Organic Frameworks (MOFs) and Second Sphere Metal-Organic Adducts

### 2.1. Coordination Polymers/Metal Organic Frameworks

**Figure 1:**
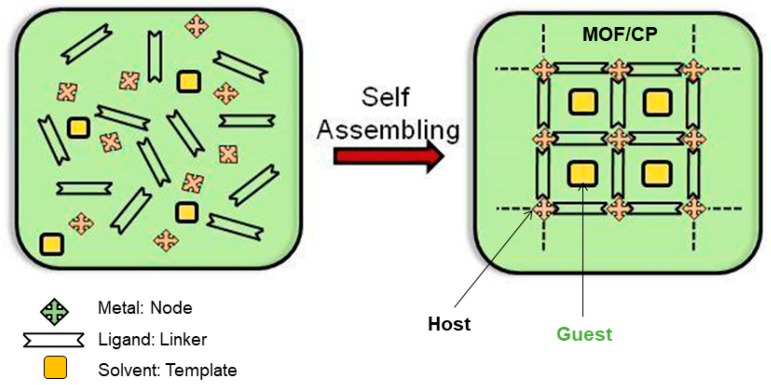
Cartoon showing the synthesis of a MOF/CP (metal organic framework/coordination polymer) by self-assembling metal ions and organic ligands in solution. Guest molecules are included in the host framework forming channels.

Coordination polymers (CPs) also known as metal organic frameworks (MOFs) are a relatively new class of hybrid metal organic materials that are built up by connecting organic molecules (linkers) with metal ions (connectors) [39,40,41,42] as shown in Figure 1. The chemistry of MOFs has developed rapidly bringing together many scientific disciplines by combining synthetic chemistry and structural analysis. Thus, a plethora of functional MOFs can now be designed using the building-block methodology by self-assembling molecular and/or ionic moieties. Determined structural topologies can be obtained by selecting ligands and metal ions with particular geometries. The resulting structures can have zero-dimensional (0D), one-dimensional (1D), two-dimensional (2D), or three-dimensional (3D) topologies. Usually the polymeric extended structures 1D to 3D are regarded as “hosts” as often contain solvent molecules trapped during the crystallization. Crucially, one important aspect for the development of MOFs is that the frameworks are stable even after the solvent (i.e., guest) is evacuated from the channels/pores, resulting in a porous material. The possibility to obtain stable porous structures boosted the research interest of MOFs materials among the scientific community, particularly for the trapping of volatile gaseous molecules for energy storage applications [43,44,45].

Depending on the topology of the host framework and on the size/volatility of the guest molecules, the final properties of the hybrid MOM can be very different and useful for many technological applications. For instance, more in a stability point of view, the dynamic behavior of the host framework can be related to the dimension of the guest molecules as they often act as templates (i.e., large guest, large channels), but also due to the flexibility of the organic linker and of the labile nature of the coordination bonds. Compared to zeolites (aluminosilicate minerals), that are considered traditional rigid porous materials, widely used in catalysis and molecular separation, CPs/MOFs are exceptional materials displaying dynamic behavior as the metal-organic framework can adapt to external stimuli by changing its structure. For instance, guest release/exchange can occur in a flexible manner, where the host framework can adapt to the new conditions (i.e., no guest or different guest) and if exposed to the original solvents can go back to the original phase. With their flexible scaffold, such dynamic MOFs have been classified as soft porous crystals [42]. Interestingly, in many cases, even after the dynamic structural transformation, the MOM framework can retain its single crystallinity, allowing full structural characterization by X-ray crystallographic methods. This aspect is crucial for the development of MOFs because X-ray crystallography can be fully exploited to study structural aspects, for instance of in situ chemical reactions happening within the host frameworks or even among included guest molecules.

### 2.2. Second Sphere Coordination Adducts

**Figure 2:**
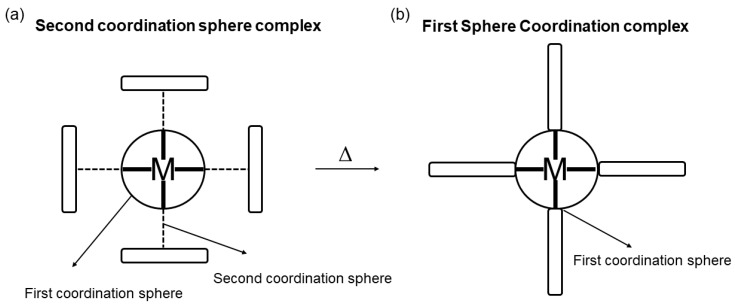
Cartoon showing the second and first coordination spheres and the transformation from the second coordination sphere (**a**) to the first coordination sphere (**b**) by means of a thermal treatment. Dark solid line indicates coordination bonds and dashed lines represent electrostatic interactions between the first and larger second sphere.

Another class of important hybrid MOMs are the so-called second sphere adducts. Second sphere adducts (i.e., also known as outer sphere adducts or “super-complexes”) are built by organic cations and metal anions self-assembled via electrostatic interactions (Figure 2a) [46,47,48]. While first sphere coordination refers to the direct interaction of ligands with metals, second sphere coordination, refers to any intermolecular interaction with the ligands directly bound to the first coordination sphere of a metal ion. Ligands belonging to the first coordination sphere can form supramolecular adducts with a wide range of noncovalent interactions such as hydrogen bonding, halogen bonding, charge transfer interactions and van der Waals interactions.

Outer sphere adducts have been focus of attention in several areas. For instance, in biochemistry [49,50], second sphere interactions were successfully used to determine activity and selectivity of certain metalloenzymes, and also in homogenous catalysis with the scope of replicating active sites found in enzymes [51]. Also, CO_2_ reduction using outer sphere biomimetic hydrogen bonds were applied in iron porphyrins [52]. In the solid-state second sphere coordination has been applied to study selective gold separation [53], gas adsorption [54], ferromagnetic behavior [55], and to build 3D and 2D perovskites as optically active materials [56].

Recently, second sphere complexes have been also studied upon heating, where the charge assisted hydrogen bonds involved in the second sphere adduct interactions are disrupted/cleaved and form new coordination bonds by dehydrochlorination reactions (Figure 2) [57]. The thermal products can be coordination complexes or in some cases extended coordination polymers. Such reactivity is in part helped by the metal–ligand reversible nature, allowing for instance, ligand exchange reactions to be studied in detail.

In the reactions where the second sphere adduct reacts to form a coordination polymer, it can be thought as a second-to-first coordination sphere transformation involving the ligand exchange with the transfer of anions from the outer sphere to the M(II) first coordination-sphere (Figure 2). In the solid-state transformation from second sphere coordination adducts to MOFs there is cleavage and formation of new chemical bonds.

Traditionally second sphere chemistry has been used to study metal-based anion receptors with direct applications in transition metal separation by direct crystallization of second sphere complexes. Further studies on the solid-state reactivity in second sphere coordination adducts is valuable because it can help in the understanding of transformations where major structural changes affecting the coordination sphere of the metal, such as chemical bond cleavage and formation occur. That chemical reactivity is crucial to obtain functional applications such as magnetism, gas adsorption or sensing and also to tailor-made new MOM with enhanced functional behavior. To the best of our knowledge, thermally induced solid-state research in the area of second sphere adducts is recent and not many examples are known in the literature.

## 3. Structural Transformations Classified upon the Degree of Crystallinity: Crystal-to-Crystal vs Crystal-to-Polycrystalline Processes

The chemistry of MOMs, particularly coordination polymers (CPs), has experienced an incredible focus of attention in the last 20 years due to their great structural versality and wide range of functional applications in areas such as catalysis, proton conductivity, gas adsorption drug delivery. One important application in CPs is the ability of explore host–guest chemistry (i.e., guest behavior) with the replacement of solvent trapped guest molecules by new incoming guests. This guest exchange has helped to develop recently the so-called crystalline sponge method reported by Fujita group, in which the crystalline structure of a material that cannot be crystallized (i.e., because is oily or cannot be obtained in large quantities to crystallize (natural products)) can be “seen” in its crystalline state within the MOF matrix [58,59]. As mentioned, the fact that the metal organic frameworks are robust yet flexible, allows guest release and exchange reactions to be carried out and to be studied structurally in detail by X-ray crystallography. In part, the huge development of MOMs is because the single crystals after a given thermal treatment can be obtained still with good quality for determining their 3D atomic arrangement by single crystal X-ray crystallography. This aspect is crucial to understand and to design a new material depending on the observed structural features before and after the solid-state reactions. That is the tailor-made design of functional materials.

**Figure 3:**
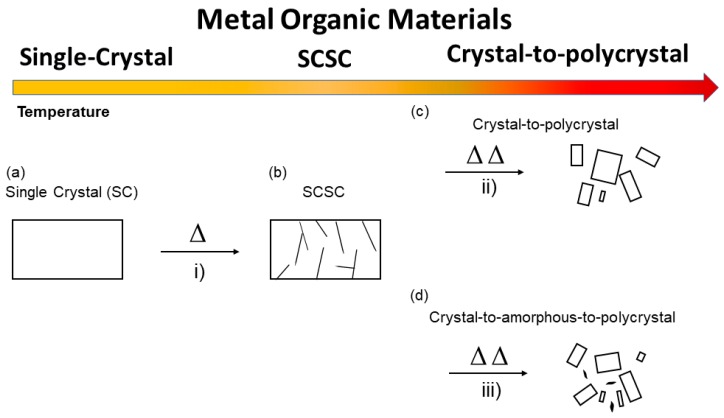
Cartoon showing the main solid-state transformations depending on the temperature discussed in the article. (**a**) Pristine single crystal that upon heating gets damaged (**b**) but still allowing single crystal XRD analysis. Further heating can lead to a crystal-to-polycrystalline transformation (**c**) or crystal to amorphous-to-polycrystalline transformation (**d**).

For the discussion of this article, it is important to distinguish three main ways (Figure 3) in which the thermal solid-state reactivity can be studied by X-ray crystallography: (i) When the reaction occurs via single-crystal-to-single-crystal manner (SCSC); (ii) when the reaction disrupts single crystallinity and forms a polycrystalline material; and (iii) when the process occurs through a (poly)crystalline-to-amorphous phase. However, it is important to note also that certain reactions in MOFs can proceed through melting and formation of glass at high temperatures. Although the importance in the development of MOFs as glasses is increasing, such processes will not be discussed in this article and the reader can find excellent examples in the recent literature [60,61].

Depending on the process that leads to the final product in the thermal reaction, in most of the cases, the structural elucidation of the material will be carried out by single crystal XRD or by powder XRD; for amorphous phases local structural information can be obtained using PDF analysis. Using a combination of such techniques a deeper understanding of a given solid-state process can shed light on the structural changes and sometimes help to understand the mechanistic aspects.

While single-crystal-to-single-crystal reactions in MOMs are quite well documented particularly within the last 10 years, crystalline-to-amorphous-to-crystalline (CAC) transformations induced by heating in hybrid metal organic materials (i.e., mostly in MOFs) are very limited. In fact, so far many of the published reports are mainly focused in structural transformations that produce amorphous phases, but only due to desolvation inducing considerable atomic motion. In such cases there is no bond breaking and/or formation. In some situations, the amorphous phases can revert-back to the original parent or a different structure upon immersion or via a gas–solid reaction in contact with the original or similar guests. Thus, such reaction can be regarded as desolvation/re-solvation process via an intermediate amorphous phase. On the other hand, some other amorphous phases do not go back to the initial or new phases but serve to create a new structure, acting as a precursor.

## 4. Thermally Induced Solid-State Reactivity in CP/MOFs

### 4.1. Thermally Induced Single-Crystal-to-Single-Crystal (SCSC) Reactions in CPs/MOFs

**Figure 4:**
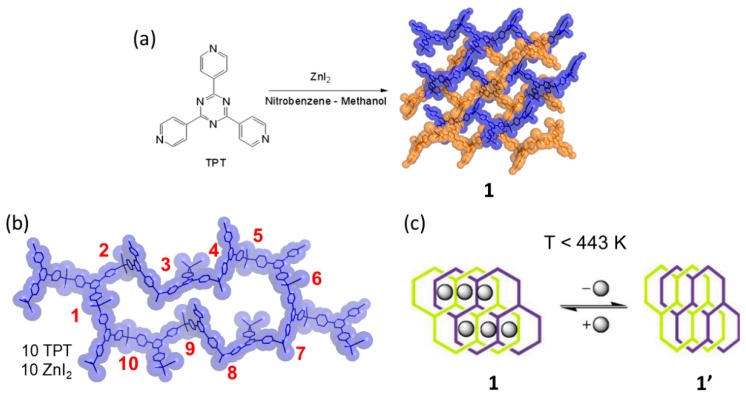
Synthesis of 1 using (tris (4-pyridyl) triazine (TPT)) ligand and ZnI_2_ (**a**). The metal organic circuit formed with 10 TPT ligands and 10 metals giving rise to the 10,3-*b* network (**b**). Desolvation process represented by a cartoon at T < 443 K (**c**). Figure 1c is reproduced from [62] with permission.

An intriguing aspect in the solid-state chemistry of CPs is to monitor the structural behavior upon external stimuli such as an increase of temperature. One of the first examples showing the potential dynamic behavior of a CP/MOF was carried out in 2002 by Fujita and co-workers using an interpenetrated CP (10,3)-*b* network self-assembled with the tridentate ligand tris (4-pyridyl) triazine (TPT) (L1) and ZnI_2_ including nitrobenzene (1) (Figure 4) [62]. The experiment was carried out heating in a hotplate up to 446 K a single crystal of 1. Due to the thermal treatment, the single crystal of 1 was still suitable for analyze it by single crystal XRD structural analysis.

The X-ray crystallographic data analysis allowed its structural solution to be determined. The X-ray structure solution revealed that the space group was changed from monoclinic (*C*2/*c*) to triclinic (*P*-1) after the guest release. But the most important aspect showed that there was an important sliding of the interpenetrated framework as a result of the guest loss. Crucially, it was reported that the solvent could be released and then re-adsorbed if exposed again to the original solvent. The process was studied by single crystal X-ray diffraction data in a SCSC process and it was observed that the interpenetrated networks could contract and expand upon guest release and uptake. The unit cell reduction upon guest evacuation was about 23% indicating a quite important compressibility of the (10,3)-*b* coordination networks and hence a surprising dynamic behavior for a system with a large unit cell (ca., 16.200 Å^3^). This article was very important as it opened up to many researchers the interest to study the dynamic behavior in CPs by using single crystal XRD analysis.

Higher level of solid-state reactivity (i.e., transformations that do not rely only on guest release and dynamic motion of frameworks), such as breaking and formation of chemical bonds, that could be monitored by single crystal XRD, were still unprecedented within MOFs.

In 2005, Chen reported a quite remarkable single-crystal-to-single crystal transformation in a CP [Ag_6_Cl(atz)_4_]OH·6H_2_O (2) which was obtained by slow evaporation of an ammonia solution of Hatz (3-amino-1,2,4-triazole) (L2) and the metal salt AgCl [63]. In 2, the Ag_3_(atz)_2_ nets are parallel to the c-axis and 5-fold interpenetrated. In a close interlocked fashion, the 5-fold chains display short inter Ag···Ag contacts (3.450 Å). The structure displays 1D channels along the *c*-axis containing disordered OH^−^ and H_2_O guest molecules.

**Figure 5:**
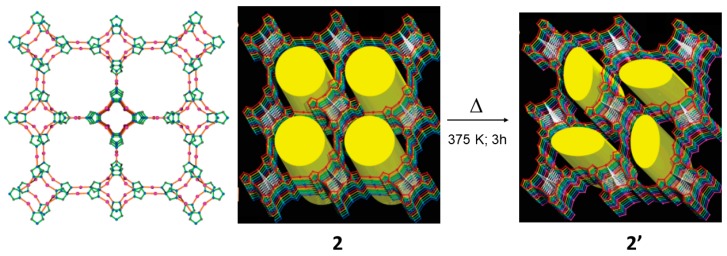
View of a single Ag_3_(atz)_2_ net (amino groups have been omitted for clarity) along the *c*-axis in 2 (left). Structure of MOF 2 showing the channels and the new structure upon SCSC reaction to form 2′. This figure is reproduced from [63] with permission.

Interestingly, upon heating the MOM to 375 K for 3 h it was possible to record data of enough quality to solve the crystal structure of the partially dehydrated phase by SC-SC XRD methods (2′). In the structure the channels (4.3 Å × 10.4 Å) were distorted compared to the ones from the original phase (2) (8.5 Å × 8.5 Å) as shown in Figure 5. Despite the considerable transformation from 2→2′ the topology of the Ag_3_(atz)_2_ net is retained with similar structural parameters of the infinite Ag_4_(μ8-Cl) chain but with the significant change that the Ag_3_(atz)_2_ net changed to 6-fold interpenetration. As a result of the severe structural transformation the void space was reduced from 32.7% to 25.2%.

Form the single crystal XRD analysis it was observed such significant change in intepenetration took place not only due to network deformation but because cleavage and formation of Ag^I^—ligand coordination bonds did occur. The original structure 2 can be obtained if 2′ is exposed to water vapor for 1 day, although only unit cell indexing was possible, thus suggesting that the change in the intepenetration from 6-fold→5-fold damaged substantially the integrity of the single crystal. The MOM display major structural transformations induced by temperature such as bond cleavage and formation to allow the changes in the network interpenetration and it was possible to be monitored by SCSC X-ray diffraction.

The solid-state reactivity in coordination polymers allows also to investigate reactions in the metal centers such as substitutions at active metal sites within the framework structures. Structural transformations involving ligand exchange might affect the *d*-orbital configuration which allows electronic and spin tuning in MOFs/CPs. Using a rod-like 1,4-bis(4-pyridil) ligand functionalized with ethylene glycol lateral chains (L3) and Co(II), Kawano and Fujita reported in 2006 an example of apical coordination transformation upon heating using the network {[Co(II)(L3)_2_(H_2_O)_2_](NO_3_)_2_·1.5(H_2_O)}*_n_* (3) [64]. The CP is a square grid-like non-interpenetrated 15.7 × 15.7 Å^2^ with the cobalt center coordinated by four pyridyl groups at equatorial positions and two water molecules at apical positions. Disordered water and two nitrate anions occupy the square cavities. The bidimensional layers are stacked on each other in an offset fashion. Thermal analysis show that all the water molecules are released in the range of 398 K–363 K. The amount of dehydrated water molecules corresponds to 6.47% of the total weight. DSC showed two endothermic peaks at 367 K and 350 K associated to the two types of water; the one coordinated to the Co and the other two as guests.

**Figure 6:**
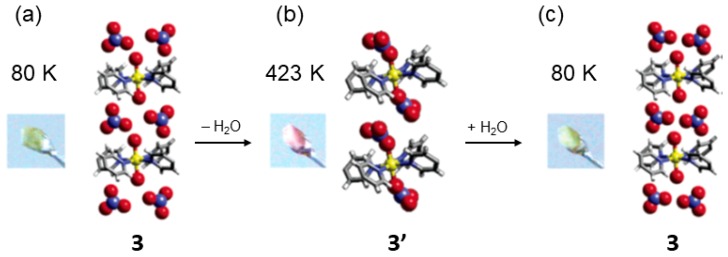
Single crystal X-ray structure of 3. Temperature dependent color change of 3 (yellow, aqua form) that upon water release transforms into 3′ (red, nitrate form). This figure is reproduced from [64] with permission.

Going to higher temperatures than those used in the DSC, crystal 3 was heated up to 473 K for 24 h (i.e., the chemical decomposition of 3 was determined at 493 K). The structural transformation occurs with a color change from yellow to red whilst maintaining the crystallinity of the material. Thus the reaction was suitable to be studied by X-ray crystallography leaving to the dehydrated coordination polymer {[Co(L3)_2_](NO_3_)_2_}*_n_*(3′) which maintained the system and space group of 3. The solid-state transformation accounts for a change of coordinated water for a NO_3_^2−^ anion at the apical position of the Co metal center. Additionally, the structural transformation is also accompanied with a framework change from cationic to neutral form after the coordination of the nitrate ions to the apical positions of the metal center (Figure 6).

The authors measured the temperature dependence of the magnetic susceptibility of 3 and 3′ which showed that the magnetic interaction between Co(II) metal centers (Weiss constant *θ*) was *θ* = 0.2 (2) K and *θ* = 0.1 (4) K for 3 and 3′ respectively. Thus, the magnetic behavior can be described as antiferromagnetic due to the interactions between closest Co(II) molecules, which is explained by the long distance among the metal centers in the crystal (i.e., ca. 15 Å and ca. 8.6 Å for the interlayer and intralayer Co atoms).

So far, the exceptional structural and chemical features reported in MOFs opened up for exploring mechanisms involving large dynamic behaviors that could give insights into more complicated structural processes. For instance, the outstanding stability in absence of guests coupled with flexible behavior gave a good prospect to study gas adsorption mechanisms in MOFs, a physico-chemical process of much importance in industry.

**Figure 7:**
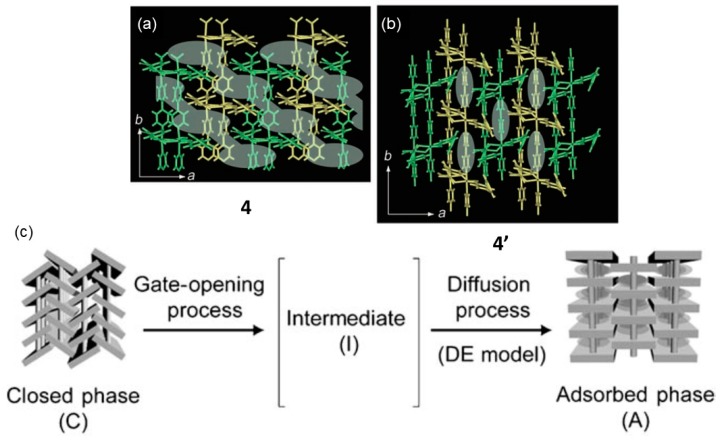
Single crystal structure of 4 (**a**) and 4′ (**b**). Cartoon showing the transition from a closed phase via an intermediate phase to give the adsorbed phase of 4 (**c**). This figure is reproduced from [65] with permission.

Kitagawa and co-workers reported in 2008 a thermally induced transformation upon guest release to produce a guest-free CP at 413 K that maintained its integrity allowing SC-XRD data to be measured [65]. The CP is built using two ligands: Benzophenone-4,4′-dicarboxylate (bpndc) and 4,4′-bipyridyl (bpy). Both ligands in the presence of Cd(NO_3_)_2_·4H_2_O in DMF resulted in the solvated framework compounds {[Cd(bpndc)(bpy)](dmf)(H_2_O)}n (1·Solvents) (4). The SC-XRD data reveled that the Cd metal centers are coordinated to bpndc to give double 1D chains expanding along the *c*-axis which are further linked via bpy ligands along the *b*-axis forming a 3D structure (Figure 7). The framework forms 1D channels and the void volume V_void_ is 29.4% of the whole crystal volume. The structure contains two DMF and two water molecules. Importantly, solvent molecules were released from room temperature to 413 K and the structure maintained its single crystallinity up to 503 K, indicating that the guest-free structure (4′) was stable. Such crystal stability allowed SCSC X-ray diffraction to be carried out. In the structural transformation the space group changed from from *C*2/*c* to *P*2_1_/*a* with a 50% reduction on the *c*-axis length. The volume reduction was 14.3% of the original phase and the V_void_ is 11.3%.

This new desolvated phase 4′ proved to be very important in order to understand the dynamic behavior in flexible porous CP. Particularly, in gaining insights on the process at which the host framework moves from a closed to an open form and starts to adsorb guest molecules (gas). The authors performed gas adsorption experiments at (90 K) using O_2_, Ar, and N_2_ showing gate-opening pressures (*P*_go_) that were increasing considerably from 3.9, 40.1, and 55.4 kPa respectively. Such huge difference in *P*_go_ was intriguing attained the small difference on the physical properties of O_2_, Ar, and N_2_. From the isotherms it was clear that there was no gas diffusion into the channels/cavities below *P*_go_. Thus, at *P*_go_ the gates of the voids/grooves in 4′ should open allowing the structure to transform into an open phase (i.e., dynamic CP).

In situ powder XRD experiments on a sample of 4′ exposed to O_2_ (4′·O_2_) were performed by LeBail analysis which showed that the lattice parameters were very similar to those of 4. This indicates that the 4′·O_2_ phase is open and expanded due to its dynamic behavior. Similar behavior was observed for 4′ exposed to Ar and N_2_. Figure 7 depicts the gate-opening model. The closed phase 4′ (C) transforms into an intermediate phase (I) that can adsorb gas molecules. Thus, the formation of the intermediate phase can be thought as the gate opening process (adsorption on the crystal surface only). Then the intermediate phase adsorbs gas forming the adsorbed form A. The thermally induced transformation via a SCSC in combination with in situ powder XRD opened up for crucial understanding of the gate-opening (GO) process in order to fine-tune gas adsorption performance of porous CPs.

In 2009 Monge and co-workers reported a thermally induced solid-state transformation using the ytterbium succinate MOF ([Yb(C_4_H_4_O_4_)_1.5_] (5) which was prepared by hydrothermal reaction of ytterbium nitrate and succinic acid [66]. The ytterbium MOF, upon heating transformed its coordination sphere, and as the authors mention changed from polymorph *α* (5) to polymorph *β* (5′) at 429 K. The authors observed the transformation using DSC and powder XRD analysis. From the powder XRD data, the indexing of the unit cell indicated that a new crystalline form was obtained. In that case full structure determination using ab initio powder XRD analysis was not attempted. Instead, the authors carried out the high temperature experiment using a single crystal in order to understand the structural changes occurred in the solid-state.

**Figure 8:**
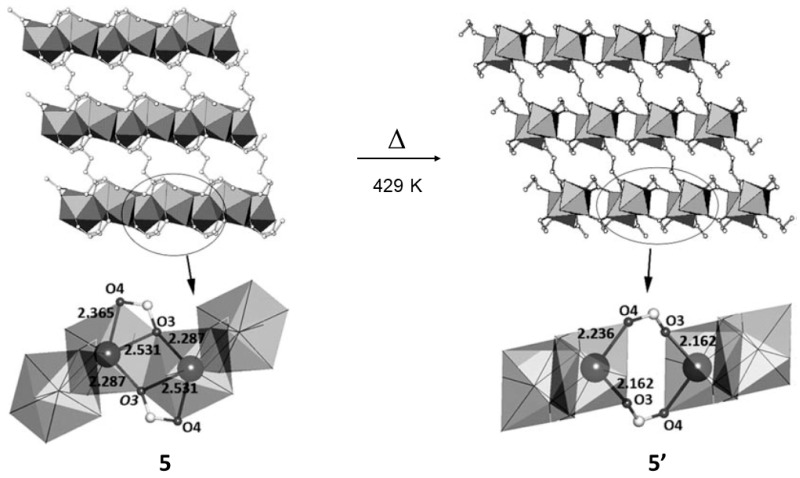
Single crystal structures of 5 and 5′. The thermal reaction induces a change in the Yb coordination sphere upon chemical bond breaking and formation. This figure is reproduced from [66] with permission.

The structural changes are in the coordination sphere of the Yb. In polymorph α the Yb forms triangular dedecahedron YbO_8_ geometry which are linked by sharing edges giving rise to zig-zag chains expanding along the *a*-axis. The chains are connected by two succinate anions along the *c*- and *b*- crystallographic directions leading a 3D MOF. The solid-sate transformation induced a change in the coordination sphere of the Yb metal giving rise to an YbO_7_ pentagonal bipyramidal geometry (Figure 8).

The combination of experimental data and theoretical calculations aiming at a better understanding of a given solid-state reaction is always useful. For instance, valuable details can be obtained on the thermodynamic stability of a certain product material after thermal reactivity and to understand if a thermally induced reaction is exothermic or endothermic according to the variation on enthalpy (Δ*H*).

**Figure 9:**
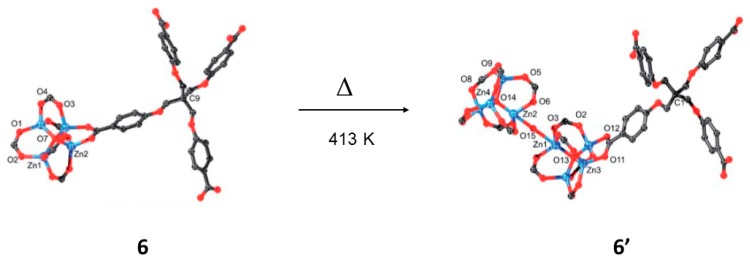
Structures showing the coordination environments of Zn_4_O(CO_2_)_6_ units in 6 (a) and 6′ (b). The transformation involves a dimerization of the Zn_4_O(CO_2_)_6_ units by a water molecule. The reaction is irreversible, and it occurs via a single-crystal-to-single crystal manner (SCSC) reaction. This figure is reproduced from [67] with permission.

In this regard, Lan and Su and co-workers reported the solid-state reactivity in which a non-interpenetrated is transformed into a self-intepenetrated MOF as a function of temperature and time [67]. The high temperature structure resulted to be air-stable compared to the unstable starting non-interpenetrated phase. For such study the ([(Zn_4_O)_2_(L)_3_·10H_2_O·46DMA) (6) and ([(Zn_4_O)_2_(L)_3_H_2_O]·H_2_O·4DMA) (6′) MOFs were used. The authors used the ligand methanetetra (tetrakis[4-(carboxyphenyl)oxamethyl]-methane acid) (H4L) for the self-assembling of the MOMs. The SCSC reported the unprecendented transformation among 3D structures via the dimerization of secondary building blocks Zn_4_O(CO_2_)_6_ clusters by means of one H_2_O molecule (Figure 9). The interconversion 6→6′ was achieved upon heating 6 at 413 K for 3 days in a sealed melting point tube. The solid-state transformation which is irreversible, is triggered by reaction temperature and time simultaneously.

Quantum mechanical calculations are very useful to gain insight in the reaction conditions and in this case by means of DFT methods selecting the sub-building units with the benzenes substituted by H atoms. The theoretical calculations at 438.15 K and standard atmosphere pressure showed that the 6→6′ process is exothermic with Δ*H* = −180.60 kcal/mol and according to the Gibbs free energy shows that the formation of 6′ is spontaneous (−51.94 kcal/mol). Thus the DFT calculations show that the reaction leads the thermodynamically favorable structure of 6′. Importantly the process was followed by single crystal X-ray in a SCSC manner.

Mechanistic aspects in heat induced solid-state such as transformations involving changes in network interpenetration, are crucial for the full understanding of the chemical reactivity and flexibility of a given MOM.

**Figure 10:**
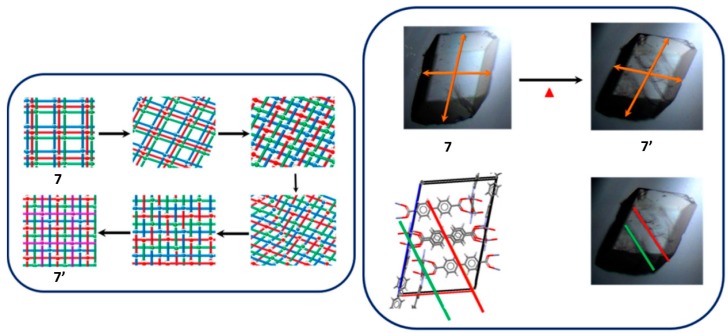
Proposed mechanism for the change in the interpenetration from 3-fold to 4-fold in 7 and 7′ respectively. Pictures taken with an optical microscope showing the macroscopic variations suffered by the single crystal upon heating. This figure is reproduced from [68] with permission.

Recently in 2018 Zaworotko and coworkers reported an interesting structural transformation focusing on the change in the level of interpenetration of the networks [68]. The network X-pcu-1-Zn-3i-α (7) transforms first to a desolvated phase at 323 K via a SCSC phase transformation X-pcu-1-Zn-3i-β (7′) with a volume reduction of 13% relative to that of the solvated structure. Further heating to 403 K forms a new phase which is 4-fold interpenetrated X-pcu-1-Zn-4i. Although the crystallinity of the sample was reduced, it was enough to solve the structure. From the SC-XRD data it was observed that the change in interpenetration is explained by the release of DMF solvent that makes the independent networks to come closer to each other, allowing the change in the interpenetration from 3-fold→4-fold via cleavage and formation of coordination bonds (Figure 10).

### 4.2. Thermally Induced Crystal to Polycrystalline Transformations in CPs/MOFs

Single crystal X-ray diffraction is the most powerful technique to determine the 3D atomic arrangement in the crystal lattice of a crystalline material. Such information is crucial if accurate knowledge on bond lengths and angles, connectivity and conformation are sought. However, SC-XRD has the limitation that a single crystal of good size and quality must be obtained. As it has been shown in the previous sections, MOFs/CPs maintain their single crystallinity even if they are affected by thermal treatment. Thus, they can be studied by single crystal XRD. However, many solid-state reactions disrupt their single crystallinity producing powdered materials. In that scenario, structure solution from powder X-ray diffraction data is the technique used to solve the crystal structures. High resolution powder XRD data (often synchrotron data) is needed to solve the structures of microcrystalline materials because often MOFs/CPs have very large unit cells, low symmetry and some degree of crystallinity is lost due to the solid-state reactivity (i.e., heating). This implies that peak overlapping is considerable and hence unit cell indexing and space group determination becomes very challenging. However, some progress has been done using ab initio powder XRD in the structural analysis of MOFs obtained upon heating.

**Figure 11:**
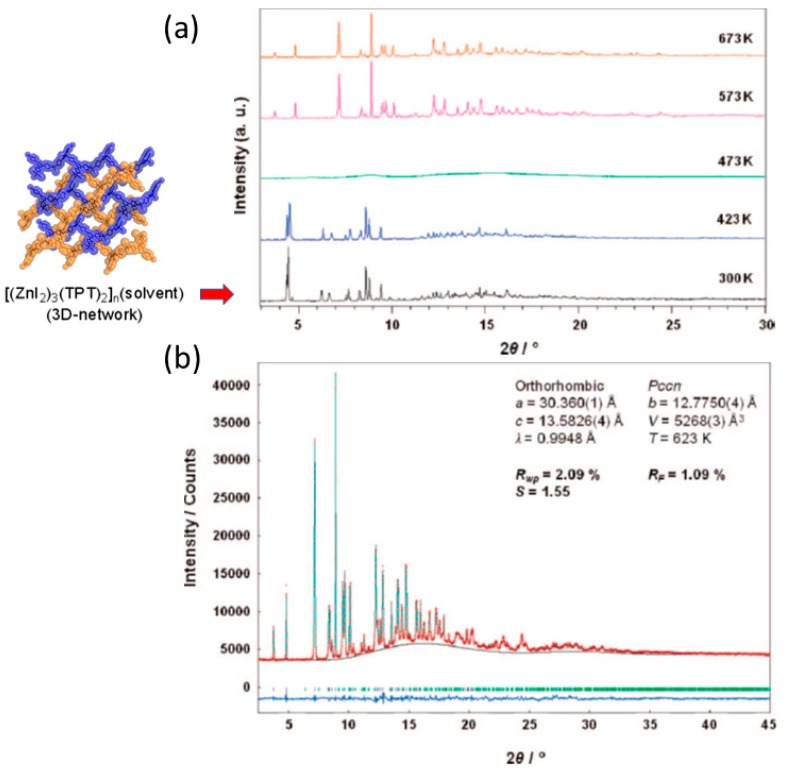
In situ synchrotron powder XRD diffractograms of 1 at different temperatures (**a**). The crystalline-to-amorphous-to-crystalline transformation is clear at 473 K which transforms further to a new crystalline phase at 573 K being stable up to 673 K. Rietveld refinement of the high temperature porous structure of 1″ (**b**). This figure is reproduced from [69] with permission.

In 2009, the (10,3)-*b* network (1) was studied under thermal effect at temperatures higher than 443 K in an attempt to explore what happen to the interpenetrated networks [69]. That is to study the framework behavior at temperatures that go beyond the SCSC limit (i.e., crystal to polycrystalline transformation). As reported, the [(ZnI_2_)_3_(TPT)_2_]*_n_*·x(solvent) forms an interpenetrated network with nitrobenzene included in the 3D channel network structure (ca., 50% voids space). In order to study 1 it was obtained as microcrystalline material upon instant synthesis (i.e., rapid mixing of ligand TPT and ZnI_2_), and in large quantities (i.e., multigram scale) which was then studied at high temperatures from 300 K to 673 K. Upon in situ heating, it was observed that after the first guest release (i.e., the first temperature range in the SCSC regime), the structural transformation was severe because the crystallinity during the reaction was not maintained, leading to an amorphous phase (473 K). Interestingly, further heating the amorphous phase transformed into a new crystalline phase (573 K) referred as 1″ (Figure 11). The amorphous phase can be “seen” for about a temperature range of 100 K. Thus, the new crystalline material 1″ was formed by means of a crystalline to amorphous phase transition (CAC).

**Figure 12:**
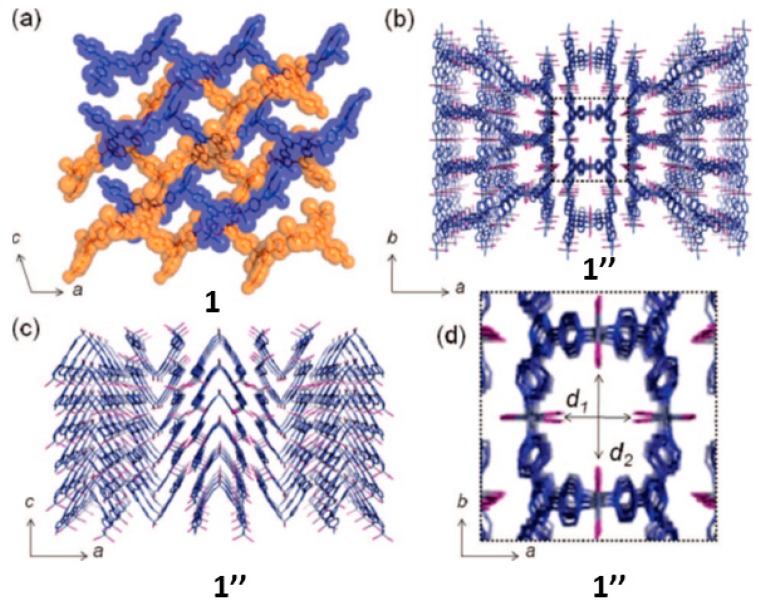
(**a**) Crystal structure of interpenetrated 1 obtained from single crystal XRD (i.e., kinetic phase). (**b**) Ab initio powder XRD structure of 1″ viewed along the 1D channels; (**c**) viewed along the 1D chain sub-structures; (**d**) detailed view of the pores. This figure is reproduced from [69] with permission.

Thus the, structure solution was carried out using high resolution synchrotron powder XRD analysis in one of the first examples of high temperature synthesis of a porous CP solved by ab initio powder XRD [70]. The structure solution was carried out employing real-space global optimization strategies (i.e., simulated annealing (SA) implemented in the DASH [55] program) followed by Rietveld refinement. The good agreement shown in the Rietveld refinement plot vindicates the correctness of the obtained structure (Figure 11). The structure was described as 1D CP forming a saddle like structure (1″) with pores (~8.3 Å × 10.5 Å) as shown in Figure 12. The thermally stable porous CP up to 643 K was able to reabsorb nitrobenzene and I_2_ from the gas phase.

In 2011, the same group carried out further studies on isostructural and non-isostructural materials of the [(ZnX_2_)_3_(TPT)_2_]*_n_*·x(solvent) where X = Cl, Br or I) revealed important insights on the framework behavior [71,72]. From such results, it was observed how the network interpenetration could be broken and form new framework structures including 1D CPs by means breaking and bond formation of coordination bonds. Also, the transformation from non-interpenetrated to interpenetrated following via an intermediate amorphous phase following a CAC transformation was reported. Importantly, the crystallization of the high temperature phase from the amorphous phase, was also observed by DSC data as and exothermic peak. The dynamic behavior of CPs was corroborated but with also a considerable reactivity induced by temperature. In this reaction it is important to consider the kinetic versus thermodynamic structures which upon heating the less stable kinetic framework can change into the more stable thermodynamic one. It was postulated that the difference in reactivity could be due to the zinc halides used involved in the framework as they are isostructural materials.

Thus, it was demonstrated that amorphous phases as intermediates, could act as precursors for the formation of new crystalline and highly porous crystalline structures. The thermally induced solid-state reactivity occurs via CAC phase transitions where the amorphous phases retained some degree of structural information (i.e., structural memory effect). Therefore, more reports involving CAC were needed for gaining insights in this type of solid-state reactions.

**Figure 13:**
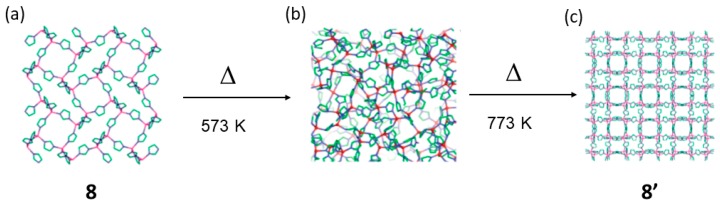
Structure of 8 (**a**), that upon heating transforms via an intermediate amorphous phase (**b**) into a new crystalline material 8′ having the zinc iodide topology (**c**). This figure has been reproduced from [73] with permission.

In 2011 Cheetham and coworkers reported a quite high temperature transformation using zeolitic imidazolate frameworks (ZIFs) implementing PDF analysis [73,74]. The framework Zn(Im)_2_ (8) includes solvent in its structure (DMF) as it is trapped during the solvothermal synthesis. Heating to about 473 K the network releases its solvent but maintains the framework structure, but upon further heating becomes amorphous at ca. 573 K with a disordered structure. Interestingly, upon further heating up to 723 K, a new crystalline structure is formed and adopts the structure of the zinc iodide (8′) (Figure 13). Similar structural transformations were observed with other ZIFs with the same composition but different zeolitic topologies. Thus, the process in this case can also be referred as crystalline-to-amorphous-to-crystalline transformation.

**Figure 14:**
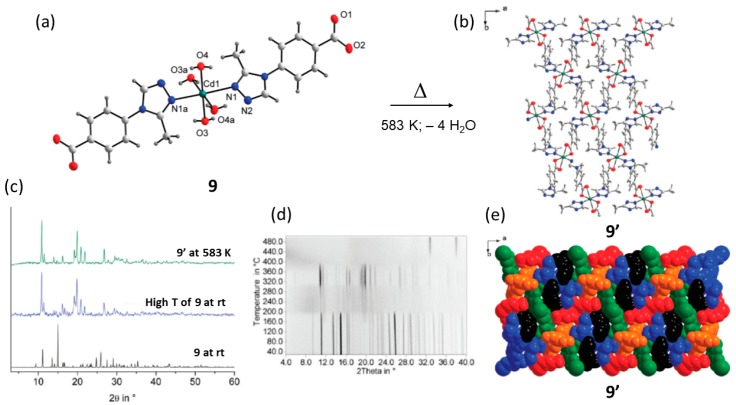
Structure of 9 showing the four water molecules coordinated to the Cd metal (**a**) which upon heating transforms into a new structure 9′ with different coordination environment (**b**). Powder XRD patterns of 9 at room temperature and 9′ (**c**). Guinier–Simon in situ powder XRD patterns at different temperatures (**d**). In space filling model, the 5-fold interpenetration is shown (**e**). This figure is reproduced from [75] with permission.

Another example of a thermal transformation with intermediate amorphous phases (i.e., CAC transformation) was reported in 2012 by Krautscheid and co-workers using two substituted 1,2,4-triazolyl benzoates (L5) they synthezed the crystal structures of a cadmium complex as well as five zinc and cadmium coordination polymers (9) [75]. Furthermore, the same group report on the thermal conversion of complexes, into crystalline and highly interpenetrated 3D coordination polymers with a high thermal stability up to 653 K. As shown in Figure 14, the cadmium complex upon heating to 473 K undergoes a dehydration process which leads to an intermediate amorphous phase that exist from 473 K to 583 K. At temperatures above 583 K, a new crystalline phase (9′) is formed and is stable up to 653 K. Further heating results in the decomposition of the material.

Like in most of the cases the high temperature phase results as microcrystalline material which cannot be analyzed by SC-XRD methods. However, a crystalline material with the same simulated powder XRD pattern can be obtained by solvothermal methods. Good quality single crystals were used for SC-XRD analysis which shows that 9′ has a completely different coordination environment around the Cd metal center and drastically diverse packing. In the 9→9′ reaction, a solid-state transformation occurs, transforming a discrete coordination complex into a 3D CP/MOFs with high degree of network interpenetration.

In this solid-state reaction upon the loss of the four coordinated water per formula unit of 9, a dramatic atomic rearrangement (ligands and metals) takes place forming the 3D network. In the process all coordinative bonds are cleaved and re-formed as observed by the coordination mode. The thermally stable crystal structure of 9′ shows no voids or channels and is tightly packed.

Using a different ligand, the authors also showed how similar transformations occur starting from 1D coordination polymers, which upon thermal treatment via and amorphous phase lead to a highly thermodynamically stable 3D network. In this case the formation of the amorphous phase is at 503 K and remains up to 563 K as a new crystalline phase is observed and is stable up to 653 K. The microcrystalline nature of the high temperature phase did not yield good quality single crystals. The crystal structure was elucidated by SC-XRD with suitable single crystals obtained by solvothermal methods.

The same group also reported how solid-state transformations using networks of higher connectivity (3D) using Zn instead of Cd metal could occur by heating in the solid-state. Interestingly, in these examples it is possible the see how different is the T range in which the amorphous phase is obtained.

As observed, the intermediate amorphous phases are intriguing as certainly their role in the process of a thermal reaction is crucial to get the high temperature phases. However, is it possible to gain structural details of the intermediate phases if they are non-crystalline? How is the structure at the local environment of the metal center?

**Figure 15:**
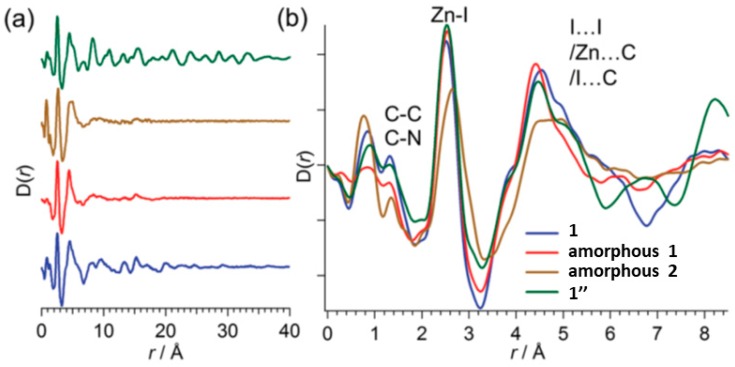
Pair distribution function (PDF) of 1, amorphous 1, amorphous 2 and 1″ (**a**). Magnification of the *r*-range showed in (**b**). This figure has been reproduced from [76] with permission.

Recently, in 2017, Ohtsu and Kawano reported a very interesting result on the crystalline-to-amorphous-to-amorphous-to-crystalline phase transition in MOF 1 [76]. The authors found that in the amorphous phase there are two type of amorphous structures. A first amorphous structure that can be transformed to the original structure that contains solvent (nitrobenzene) which is followed by a second amorphous structure which cannot be reversibly transformed into the original phase 1, but that upon heating forms the thermally stable 1D porous crystalline structure 1″. Insights into the structural details were analyzed by X-ray absorption fine structure (XAFS) and X-ray pair distribution function (PDF). From the XAFS experiments it was determined that the tetrahedral geometry on the Zn metal was maintained in the reversible amorphous phase and in the porous 1D 1″. The PDFs of phase 1 and 1″ showed long range ordering due to their crystalline nature. However, for the intermediate amorphous phases, only short-range order was observed with intensity decreasing at high *r* (Figure 15). Whereas PDFs of 1, first amorphous and 1″ are almost identical which suggest the same regular Zn coordination environment, the PDF of the second amorphous phase indicates a distorted Zn coordination environments appear to be key intermediate phase for the crystallization of the 1D 1″ porous and stable MOF.

## 5. Thermally Induced Solid-State Reactivity in SSC

### 5.1. Thermally Induced Single-Crystal-to-Single-Crystal (SCSC) Reactions in SSCs

Second sphere adducts, being MOMs with transition metals and organic molecules in their structures, can be used to study their solid-state reactivity. In fact, thermal treatment can be used to generate different second sphere adducts by means of dehydrochlorination reactions. In the reaction the release of HCl from the second sphere complex can lead in the formation of new compounds with the same dimensionality (0D) of new MOMs with higher dimensionality such as 1D coordination polymers. In some cases the reactivity can be reversible by applying chemisorption reactions usually with HX (where X is usually Cl or Br) via gas solid processes.

Orpen in 2005 reported the first example of a dehydrochlorination reaction of a second sphere adduct that transformed into a neutral complex upon mechanochemical grinding in the presence of a strong base (KOH) [77]. One drawback of using mechanochemical methods is that KCl forms as a by-product which sometimes can be an impediment as purification has to be carried out if a pure product has to be used. If instead of mechanochemical, thermal treatment is used, the formation of the by-product is avoided. Thus, in 2007 a dehydrochlorination reaction was carried out by Orpen and coworkers upon heating a charge assisted hydrogen bond second sphere complex, which resulted in a neutral complex [57]. It is important to note that in some second sphere adducts the release of HCl can occur spontaneously in contact with the atmosphere following a crystalline to polycrystalline transformation [78]. In this case heat is not needed for the structural transformation and the structure solution was carried out by ab initio powder XRD analysis. Importantly, to the best of our knowledge, transformations that upon heating result in the formation of CPs (mainly 1D CPs) are quite rare. Nevertheless, there are some cases that deserve to be highlighted. SCSC reactions induced by temperature in second sphere adducts which are much less reported that SCSC in MOFs are also intriguing.

**Figure 16:**
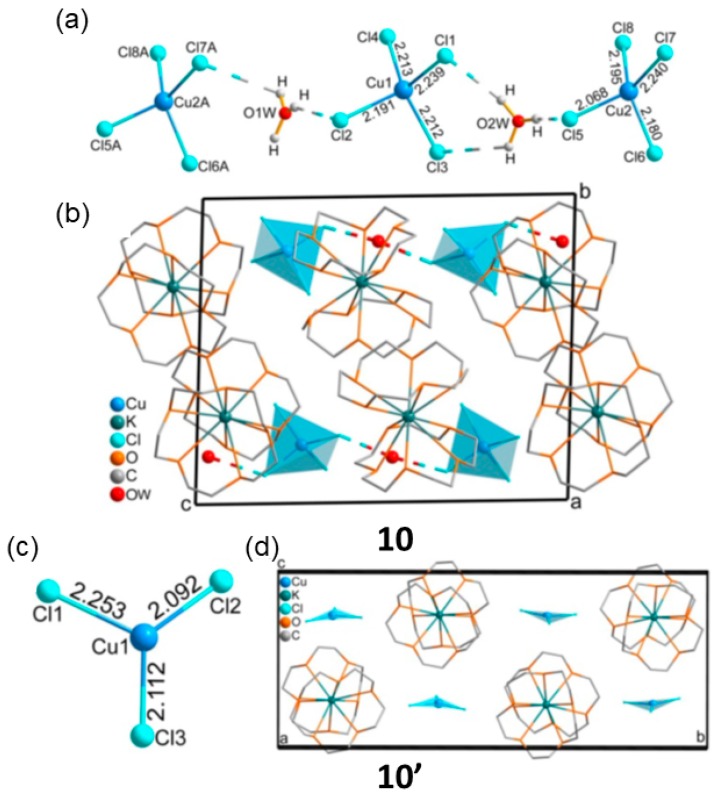
Structure of 10 showing the bridging H_3_O^+^ cations and the [CuCl_4_]^2−^ dianions (**a**) and crystal packing (**b**). Crystal structure of 10′ showing the rare [CuCl_3_]^−^ monoanion (**c**) and crystal packing (**d**). This figure is reproduced from [79] with permission.

Guo and Wang in 2017 using a discrete non-porous second sphere adduct (H_3_O)[K(15-crown-5)_2_][CuCl_4_] (10) showed how the adduct changed its magnetic properties upon structural modification by means of a chemisorption reaction [79]. The notable fact is that the reaction occurs in a SCSC manner following a thermally induced desorption of H_2_O and HCl that can be reverted back to the original material by vapor resorption of H_2_O and HCl. The process has been corroborated also using microcrystalline samples by powder XRD. The structural changes also involved variation in the coordination number, space group and in the color sample. The asymmetric unit of 10′ contains two isolated [CuCl_4_]^2−^ anions, two [K(15-crown-5)_2_]^+^ cations and two hydrated protons. The two Cu atoms are tetracoordinated by four Cl atoms in a distorted tetrahedral geometry and linked via a H_3_O^+^ cation by means of O-H···Cl hydrogen bonds (Figure 16a).

Upon heating, as demonstrated by DSC analysis, first the H_2_O are released followed by a HCl release. Annealing the sample at 373 K for 30 min, the green sample changed color to red indicating a change in the coordination sphere of the metal. Single crystal X-ray analysis revealed that the Cu^II^ metal is coordinated by three Cl ligands in a planar geometry to give the not very common [CuCl_3_]^−^ monoanion. Thus, in the transformation [CuCl_4_]^2−^→[CuCl_3_]^−^ (10→10′) cleavage of Cu-Cl coordination bonds took place as well as the release of [H_3_O]^+^ cations from the structure of 10 to give a new discrete second sphere adduct 10′ (Figure 16b,c). Exposing the [CuCl_3_]^−^ adduct to HCl for 30 min forms the green crystals of 10 also following a SCSC reaction.

Importantly, the structural transformation has a direct influence in the magnetic properties of 10 and 10′. The presence of protonated water allows to stablish Cu-Cl–···H_3_O^+^···–Cl-Cu charge assisted hydrogen bonding interactions allowing weak antiferromagnetic behavior among Cu···Cu metal centers. Whereas in 10′, the near zero Weiss constant *θ* indicates that there is a negligible magnetic interaction among the Cu^II^ ions. The authors explain that effect due to the release of bridging protonated water, that upon heating leave the Cu metal centers too far away from each other (9.0971 Å), rendering the material paramagnetic.

This is a good example where it shows the correlation between structure and function in a hybrid MOM. Despite being non-porous, shows that upon heating the hydrated material bond breaking occurs to give a paramagnetic MOM. It also demonstrates that the second sphere adducts are quite reactive and able to change the second sphere coordination number from 4 to 3 and vice versa. Other examples where the changes involve the second to first coordination sphere are also observed in other MOMs as explained below.

**Figure 17:**
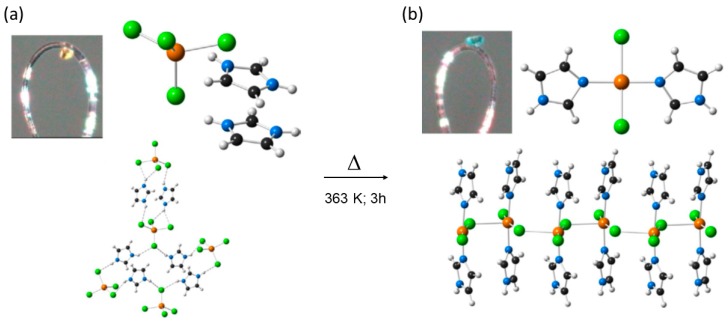
Crystal structure of the second sphere adduct 11 (**a**). Formation of a new coordination environment on 11′ upon heating with small changes in the magnetic behavior (**b**). Both materials show weak antiferromagnetic behavior. This figure has been reproduced from [80] with permission.

Another example of non-porous materials using a second sphere adduct displaying dynamic behavior upon the release of HCl molecules have been reported by SCSC. Recently, Minguez Espallargas using imidazole and CuCl_2_·2(H_2_O) reported two-consecutive magneto-structural transformations in non-porous materials with a first transformation of a polymeric-to-molecular material (i.e., second sphere adduct) followed by a second structural transformation induced by thermal treatment resulting in a 1D polymeric chain Figure 17 [80].

The 1D polymeric structures are [Cu(im)(Cl)(imH_2_)] (11) where the metal centers display a square pyramidal penta-coordinated structure with an apical Cl atom and a basal plane formed by four N atoms. The 1D CP chains can be disrupted exposing it to HCl vapors as a clear color change from blue to yellow occurs within 0.5 h. SC-XRD allowed unambiguously to determine the structure of the new phase which resulted to be a nonporous second sphere adduct of distorted tetrahedral [CuCl_4_]^2−^ dianions and [imH_2_]^+^ cations. By thermal treatment at 363 K for 3 h, the second sphere adduct can be transformed to a new 1D CP (11′) following the release of HCl upon bond Cu-Cl and N-H cleavage and formation of Cu-N coordination bonds. The disruption and formation of chemical bonds can occur also spontaneously but with longer periods of time (i.e., several weeks). The new 1D CP is formed by neutral trans-[CuCl_2_(imH)_2_] units after deprotonation of the imidazolium cations in the second sphere adduct to form the neutral ligands which are further coordinated by the CuCl_2_. The coordination geometry is square planar with two nitrogen atoms from imidazolium and two chloride ligands. The 1D chain is formed by the apical interaction of on Cl ligand with the Cu atom of an adjacent chain and extends along the *c*-axis.

This solid-state reaction has a direct influence into the magnetic behavior of this 1D CP. Due to the small size of the imidazole ligands, the interexchange of magnetic properties occurs in the CPs but not in the second sphere adducts because the metal centers are too far away to allow magnetic exchange among Cu atoms. In that case the Cu-Cl···Cl-Cu interactions allow only weak antiferromagnetic behavior. However, in the 1D polymeric structure the electronic configuration is different to the one with the Cl^−^ bridging group replacing the imidazolate. Importantly, the geometry of the bridging ligand does not facilitate efficient magnetic exchange because the magnetic orbitals are almost perpendicular. Thus, the magnetic coupling in 11′ is similar to the second sphere adduct 11 than in the CP.

### 5.2. Thermally Induced Crystal to Polycrystalline Transformations in SSCs

**Figure 18:**
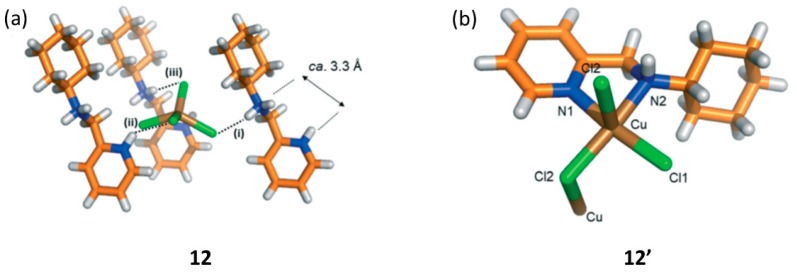
Structure of 12 (**a**) showing the hydrogen bonding interactions among cation and anion. Single crystal X-ray structure of 12′ showing the bridging coordination bond among Cu metal centers (**b**). This figure has been reproduced from [81] with permission.

Using the ditopic oily ligand N-(pyridin-2-ylmethyl) cyclohexanamine following a first diprotonation of the two N atoms (addition of HCl and salt formation), was further self-assembled with the [CuCl_4_]^2−^ dianion in order to generate a hybrid MOM (i.e., second sphere adduct) [2HL]^2+^·[CuCl_4_]^2−^ (12) [81]. In this reaction, the outer sphere was that of the dication interacting via charge assisted hydrogen bonding interactions with the [CuCl_4_]^2−^ dianion. Yellow microcrystalline sample (12) was treated thermally by annealing at 433 K for 2h in the oven. Macroscopically it was observed that the sample changed from yellow to blue, indicating that a change in the coordination sphere should have occurred as such color change is common for this type of structural transformations (Figure 18).

**Figure 19:**
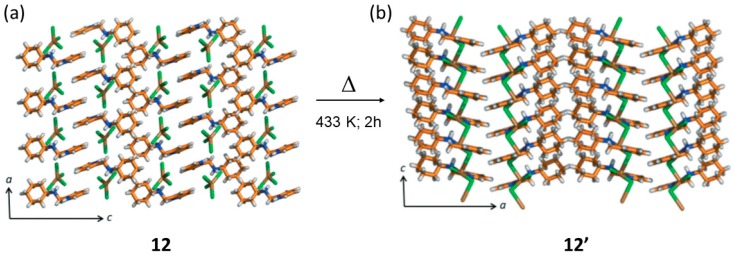
Structure of 12 (**a**) and thermal transformation via dehydrochlorination reaction to give the 1D coordination polymer 12′ (**b**). This figure has been reproduced from [81] with permission.

From the powder XRD experiments, it was observed that the obtained thermal product was different from that of the starting outer sphere adduct, and also different from the protonated chlorinated salt. In this case, the solid-state reaction was too severe that did not allow to monitor the process by SCSC. The recrystallized blue product resulted to be a 1D coordination polymer LCuCl_2_ (12′) with a bridging Cl atom. The solid-state reaction involved the release of two molecules of HCl (per formula unit) and two H atoms from the protonated ligand. The coordination geometry of the Cu(II) metal center has changed from distorted tetrahedral to a chelated non-planar penta-coordinated member ring. Viewed along the *b*-crystallographic direction the structures of 12 and 12′ are quite similar, and it can be observed that the polymeric direction in 12′ is along the *c*-axis due to the bridging Cl-*μ*-Cl bond (Figure 19).

In the solid-state transformation, there is also a charge in the electronic nature of the MOMs materials from charged in 12 to neutral in 12′. Nevertheless, under the experimental conditions, the reaction did not proceed via SCSC. Crucially, despite being non-porous if exposed to vapors of HCl, 12′ can revert back to the original structure where the second sphere interactions control the self-assembling process of the anions and cations. Although not analyzed in 12 and 12′, it could be that due to the closer vicinity of the metal centers in 12′ the magnetic properties of 12′ could be enhanced facilitating magnetic exchange among Cu atoms providing of a good orientation of magnetic orbitals.

**Figure 20:**
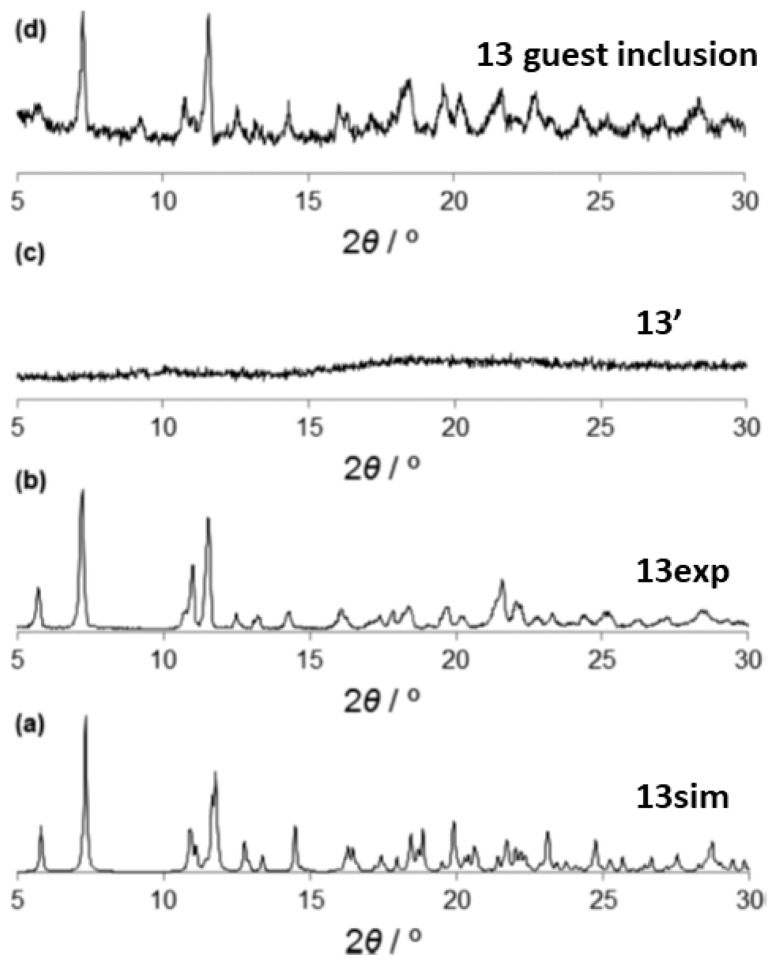
Powder XRD patterns of 13 simulated (**a**), experimental (**b**) and amorphous phase 13′ (**c**). The amorphous phase regenerates its crystalline state upon immersion in ethanol (**d**). This figure has been reproduced from [82] with permission.

An example of CAC transformation involving a second sphere adduct was reported recently. In such report the original second coordination sphere adduct self-assembled using a flexible bidentate ligand L with a long−(CH_2_)_4_−backbone chain allowing high conformational flexibility [82]. Using second sphere interactions, the double protonated ligand was self-assembled with the [HgCl_4_]^2−^ dianion as a metal center. The outer sphere adduct (13) also contained ethanol and water as guest molecules. The thermal stability was monitored by heating the hybrid metal organic adduct up to 373 K for 1h. The guest release was corroborated by ^1^H NMR and the powder XRD pattern clearly showed that an amorphous phase was obtained 13′ (Figure 20c), as a result of the structural transformation and the loss of long-range periodicity. However, the original phase with water and ethanol can be regenerated by immersion in the original solvent (EtOH). This is one of the first cases of such crystalline-to-amorphous-to-crystalline phase transition in second sphere adducts. The re-formation of the original framework indicates that a certain structural memory effect remains in the amorphous phase and proofs that the second sphere coordination adducts are flexible and adaptable to external stimuli.

## 6. Beyond the Limits of Single-Crystal-to-Single-Crystal Transformations in Hybrid Metal Organic Materials

As it has been observed, the type of reactivity undergone by the MOM will influence notably the X-ray technique that will be used for its structural characterization. In general, the higher the temperature reached in the solid-state reaction, the more severe will be the damage that the single crystal will suffer and the more likely the SCSC limit will be achieved. Table 1 summarizes the examples given in this article. It has been shown how combining single crystal XRD and ab initio powder XRD, that is going beyond the limits of single-crystal-to-single-crystal transformations, detailed information on the structural transformation can be established.

In this article the lowest temperature for a thermal process described is 363 K which corresponds to a dehydrochlorination reaction in a second sphere coordination. The solid-state transformation involves the cleavage and formation of new chemical bonds to give a 1D CP via a SCSC reaction. Thus, the solid-state transformation can be regarded as chemical reactivity in the solid-state of a MOM. Whereas the highest temperature process described herein is 733 K, is a crystallization of a MOF from an amorphous phase in zeolitic imidazolate frameworks. The process occurs following a CAC transformation with an intermediate amorphous phase. Comparison with the experimental against the simulated powder XRD pattern demonstrates that the high temperature product has the same structure of the known zinc iodide. However, for thermal process that lead to unknown structures such as in the 1→1″ transformation at 573 K, to gain structural information of 1″ needs full structure determination from ab initio powder XRD which is a non-trivial process. Despite the difficulties of solving structures from powder data, it has been observed that crucial structural information can be obtained, helping in the deeper understanding of the solid-state reactivity in MOMs, such as network transformation from interpenetrated to non-interpenetrated and vice versa. Even more, for samples that are amorphous such as the two amorphous phases observed in the CAAC in the 1→1″ transformation, it is possible to obtain information on the local environment around the metal center and hence determine the distortion on zinc-halide chemical bonds using PDF analysis. That level of structural information using high resolution synchrotron X-ray diffraction techniques is unique and shows the potential of using the latest advances on experimental approaches for establishing structural properties in hybrid metal organic materials.

The lack of a single crystal suitable for single crystal XRD has been demonstrated that can be tackled using alternative/complementary techniques able to allow structure elucidation to be carried out for powdered crystalline and non-crystalline materials. Thus, a wide range of temperatures (363 K–733 K) studying solid-state reactivity in MOMs can be monitored in order to discover new and unique phenomena this fascinating class of materials.

## 7. Conclusions

Temperature induced solid-state reactions in MOMs are very important to explore chemical reactivity that conventional solution chemistry does not allow. A good understanding on high temperature stability and reactivity in MOMs is crucial to understand the potential and the limits of their applications. The combination of structural variability, network topology, porosity, coordination bond lability and high temperature in MOMs is contributing to the discovery of important structural transformations in MOMs. The availability of single crystals of enough quality to be studied by single crystal XRD after a given thermal process is crucial for understanding the thermal products. In that case SCSC reactions are fundamental for the determining the 3D structural arrangement of the products. While powder XRD is commonly used to monitor crystallinity, stability upon guest release/inclusion, phase purity, it is difficult to find reports of structure elucidation of MOFs, particularly products obtained by heat treatment. Due to the increasing interest in the solid-state chemistry of MOFs (MOMs) across many different scientific areas, the ability to solve the crystal structures for those situations in which the synthesis of suitable crystals for single crystal XRD is of utmost importance. Thus, whenever the solid-state reactivity is too severe that disrupts single crystallinity, ab initio powder XRD has demonstrated that is a very powerful technique to elucidate the crystal structures of thermal products. In that scenario, the process can be considered as solid-state reactivity beyond single-crystal-to-single-crystal limit. Such thermally induced reactions usually involve network displacements (expansion-contraction) but also chemical reactions such coordination bond breaking/formation necessary for large changes in framework interpenetration. Another important type of heat induced processes are dehydrochlorination reactions described in second sphere adducts where chemical bonds are broken and formed usually implying loss of single crystallinity. In the last 15 years there have been many impressive reports on SCSC transformations induced by heating which have helped to gain several important aspects in the MOMs chemistry. However, there are still few fully determined structures from ab initio powder XRD where the solid-state heating products are obtained as microcrystalline materials. Clearly the combination of single crystal and powder XRD techniques is of fundamental importance for the advance in the structural knowledge of thermal MOM products. Finally, the combination of experimental data on heat induced solid-state transformations and theoretical calculations, such as quantum mechanics for solid-state systems, can be very helpful in gaining insights on such solid-state processes. Although some examples have been shown, more combination experimental-theoretical calculations should be carried out.

## Figures and Tables

**Table 1:** Thermal reactions in metal organic materials (MOMs) showing the type of material, the solid-state process, temperature and the main structural transformation described.

Starting MOM	Thermal Product	Process	Temperature	Structural Transformation	References
**1**	**1′**	SCSC^1^	446 K	Guest release, network sliding.	[62]
**1′**	**1″**	SC-PC^2^	573 K	Interpenetrated to non-intepenetrated networks; CAC^3^; bond cleavage and formation.	[69,71,72]
**2**	**2′**	SCSC	375 K	5-fold→6-fold intepenetration; bond cleavage and formation.	[63]
**3**	**3′**	SCSC	473 K	Apical H_2_O for NO_3_^2−^ ligand exchange; cationic→neutral network change.	[64]
**4**	**4′**	SCSC	413 K	Guest release, network sliding; gate opening behavior.	[65]
**5**	**5′**	SCSC	429 K	Bond breaking and formation; change from triangular dodecahedron YbO_8_ to pentagonal bipyramidal YbO_7_.	[66]
**6**	**6′**	SCSC	438 K	Non-intepenetrated to interpenetrated nets; dimerization of secondary building blocks via H_2_O; bond formation.	[67]
**7**	**7′**	SCSC	403 K	Bond cleavage and formation; 3-fold→4-fold interpenetration.	[68]
**8**	**8′**	SCPC	573 K–733 K	CAC; bond-cleavage and formation.	[73,74]
**9**	**9′**	SCPC	506 K–583 K	Discrete (0D) complex→3D CPs; 1D→3D CPs; 3D→3D CPs CAC; bond cleavage and formation.	[75]
**10**	**10′**	SCSC	373 K	Second sphere adduct→second sphere adduct; chemisorption of HCl, bond-cleavage and formation.	[79]
**11**	**11′**	SCSC	363 K	Second sphere adduct→1D CP; chemisorption of HCl bond-cleavage and formation.	[80]
**12**	**12′**	SCPC	423 K	Second sphere adduct→1D CP; chemisorption of HCl bond-cleavage and formation.	[81]
**13**	**13′**	SCPC	373 K	Second sphere adduct guest release; CAC.	[82]

^1^ SCSC: single-crystal-to-single-crystal transformation; SCPC: single-crystal-to-polycrystalline transformation; CAC: crystalline-to-amorphous-to-crystalline transformation.
